# Implementation of a Home-Based mHealth App Intervention Program With Human Mediation for Swallowing Tongue Pressure Strengthening Exercises in Older Adults: Longitudinal Observational Study

**DOI:** 10.2196/22080

**Published:** 2020-10-16

**Authors:** HyangHee Kim, Nam-Bin Cho, Jinwon Kim, Kyung Min Kim, Minji Kang, Younggeun Choi, Minjae Kim, Heecheon You, Seok In Nam, Soyeon Shin

**Affiliations:** 1 Graduate Program in Speech-Language Pathology Yonsei University College of Medicine Seoul Republic of Korea; 2 Department and Research Institute of Rehabilitation Medicine Yonsei University College of Medicine Seoul Republic of Korea; 3 Department of Industrial and Management Engineering Pohang University of Science and Technology Pohang Republic of Korea; 4 Graduate School of Social Welfare Yonsei University Seoul Republic of Korea

**Keywords:** mHealth, older adults, swallowing tongue pressure, Iowa Oral Performance Instrument, app, swallowing disorders, swallowing maneuver, effortful prolonged swallowing, effortful pitch glide, effortful tongue rotation

## Abstract

**Background:**

Tongue pressure is an effective index of swallowing function, and it decreases with aging and disease progression. Previous research has shown beneficial effects of swallowing exercises combined with myofunctional tongue-strengthening therapy on tongue function. Tongue exercises delivered through mobile health (mHealth) technologies have the potential to advance health care in the digital age to be more efficient for people with limited resources, especially older adults.

**Objective:**

The purpose of this study is to explore the immediate and long-term maintenance effects of an 8-week home-based mHealth app intervention with biweekly (ie, every 2 weeks) human mediation aimed at improving the swallowing tongue pressure in older adults.

**Methods:**

We developed an mHealth app intervention that was used for 8 weeks (3 times/day, 5 days/week, for a total of 120 sessions) by 11 community-dwelling older adults (10 women; mean age 75.7 years) who complained of swallowing difficulties. The app included a swallowing monitoring and intervention protocol with 3 therapy maneuvers: effortful prolonged swallowing, effortful pitch glide, and effortful tongue rotation. The 8-week intervention was mediated by biweekly face-to-face meetings to monitor each participant’s progress and ability to implement the training sessions according to the given protocol. Preintervention and postintervention isometric and swallowing tongue pressures were measured using the Iowa Oral Performance Instrument. We also investigated the maintenance effects of the intervention on swallowing tongue pressure at 12 weeks postintervention.

**Results:**

Of the 11 participants, 8 adhered to the home-based 8-week app therapy program with the optimal intervention dosage. At the main trial end point (ie, 8 weeks) of the intervention program, the participants demonstrated a significant increase in swallowing tongue pressure (median 17.5 kPa before the intervention and 26.5 kPa after the intervention; *P*=.046). However, long-term maintenance effects of the training program on swallowing tongue pressure at 12 weeks postintervention were not observed.

**Conclusions:**

Swallowing tongue pressure is known to be closely related to dysphagia symptoms. This is the first study to demonstrate the effectiveness of the combined methods of effortful prolonged swallowing, effortful pitch glide, and effortful tongue rotation using mobile app training accompanied by biweekly human mediation in improving swallowing tongue pressure in older adults. The mHealth app is a promising platform that can be used to deliver effective and convenient therapeutic service to vulnerable older adults. To investigate the therapeutic efficacy with a larger sample size and observe the long-term effects of the intervention program, further studies are warranted.

**International Registered Report Identifier (IRRID):**

RR2-10.2196/19585

## Introduction

The tongue plays an important role in both the oral and pharyngeal stages of swallowing, especially in proper bolus formation and propulsion [1]. Thus, its impairment is heavily linked to swallowing difficulties or dysphagia by inhibiting adequate bolus formation and transportation within the oral and pharyngeal cavities [2]. During liquid consumption, the bolus is initially held in the anterior part of the mouth or in the chamber between the tongue surface and the hard palate.

In older adults, sarcopenia, which is defined as loss of muscle mass due to aging, contributes to lingual dysfunction, resulting in swallowing impairments [3]. Previous studies have shown that sarcopenia and tongue muscle degeneration are highly related to aging [3-5] and negatively influence tongue pressure generation and swallowing in older adults, escalating the risk of aspiration and dysphagia [3,4,6].

The ability to generate tongue pressure is critical for liquid bolus propulsion in swallowing and is also an effective index for evaluating swallowing function [7,8]. Tongue pressure commonly decreases with aging [2,4], partly due to sarcopenia of the tongue [1,4]. Specifically, reduction in tongue pressure in older adults might cause an increase in oropharyngeal residue and consequently increase the risk of aspiration and pneumonia [7,9] due to reduction in proper bolus control and transfer [1]. Approximately 13% to 54% of older individuals have reported swallowing difficulties, with the rate varying with age, underlying diseases, and care level [10-12]. Major swallowing symptoms, such as coughing, choking [11,13], and malnourishment in older adults [6], are reportedly influenced by tongue dysfunction and tongue pressure measures [14-16].

Swallowing tongue pressure is generated when an individual swallows different types of food or liquid or during dry swallowing. The decrease in swallowing tongue pressure in older adults is prevalent [7,17] depending on the types and the viscosity of the intake [1,7,17,18] and during dry swallowing [7]. As the decline in tongue pressure due to aging may be indicative of risk factors related to dysphagia [17], it is crucial to pay attention to swallowing tongue pressure measures in order to incorporate swallowing rehabilitation.

Past studies have shown beneficial effects of swallowing exercises combined with myofunctional tongue-strengthening exercises in older adults, as well as in adults with various types of disease. Among diverse rehabilitative techniques using swallowing interventions, the Mendelsohn maneuver [19] and effortful swallowing are swallowing-focused methods. The implementation of both treatment methods has shown a positive effect on tongue pressure [20], as well as on pharyngeal [21] and hyolaryngeal functions [22]. Moreover, the combination of the two methods has led to increased anterior and posterior tongue pressure [23] and decreased aspiration rates in dysphagic patients [24].

Meanwhile, indirect methods of swallowing treatment include effortful pitch glide and effortful tongue rotation. Effortful pitch glide does not invoke the principle of task specificity for swallowing [25,26]. However, when effortful pitch glide was combined with the Mendelsohn maneuver, its positive effect on the activation of suprahyoid muscles, the functioning of which is regarded as essential in hyolaryngeal excursion (and thus in swallowing), was observed [27]. Such results may also account for the observed coordination of suprahyoid muscle activity, hyoid movement, and tongue pressure production during tongue squeezing [28]. A significant increase in maximum tongue pressure was also observed after effortful tongue rotation [29] and directional exercises of the tongue (ie, elevation, protrusion, and lateralization) [30].

Repetition and intensity of training are key components needed to facilitate experience-dependent neural plasticity in motor behaviors [25] and swallowing [26]. Training with evident goals, however, requires participants to regularly visit swallowing clinics. Several studies have provided information on factors associated with the difficulty of ensuring regular visits to clinics among older adults, including expenses, distance, transportation, and the time required [31,32]. Therefore, mobile health (mHealth) technologies may have great potential in advancing health care in the digital age to be more efficient for people [33] with limited resources, especially for older adults.

Thus, this study explores the immediate and long-term maintenance effects of an 8-week home-based mHealth app intervention program with human mediation aimed at improving the swallowing tongue pressure in older adults. We further highlight several clinical issues raised during the intervention program that needed to be addressed. This study was part of an ongoing larger 7-year national grant project entitled “Development and Implementation of Swallowing Evaluation and Intervention Program for Older Adults.”

## Methods

### Participants

The participants in this study were identical to those who took part in the usability study of the mHealth app [34]. The data for the usability evaluation [34] and the current study (ie, swallowing training results) were collected from these participants in the same time frame. A total of 11 older adults (10 women; mean age 75.7 years, SD 4.96; mean education 10.4 years, SD 3.83; mean Korean Mini-Mental State Examination score 28.1, SD 1.72) from 2 district-run senior welfare centers in Seoul participated in the program. The inclusion criteria were (1) older than 65 years; (2) complaints of swallowing difficulties (ie, increased aspiration rate and foreign body sensation in throat); (3) a Korean Mini-Mental State Examination score within normal limits; (4) no problem with vision, hearing, and motor functions required for using a tablet; and (5) no hearing problems that may prevent the participant from following directions. The exclusion criteria were (1) any history of neurological disorders (ie, stroke and Parkinson disease) and (2) nonoral feeding (ie, nasogastric tube and percutaneous endoscopic gastrostomy). Self-reported swallowing rating was indicated as responses to the survey item “I have a swallowing problem” (with a scale of 0=never, 1=seldom, 2=sometimes, 3=often, and 4=always). Table 1 presents the characteristics of the participants. All participants gave informed, written consent as per the Declaration of Helsinki before conducting the study (institutional review board No. PIRB-2019-E024).

**Table 1:** Participant characteristics.

Participant No.	Gender	Age (years)	Education (years)	K-MMSE^a^ score	Self-reported swallowing rating^b^
1	Female	80	6	30	3
2	Female	71	16	29	3
3	Female	67	12	30	2
4	Female	83	9	24	4
5	Female	77	12	27	3
6	Female	71	9	29	2
7	Female	82	6	29	3
8	Female	73	12	28	3
9	Female	76	5	27	2
10	Male	75	16	28	2
11	Female	78	6	29	2
Total, mean (SD)	N/A^c^	75.73 (4.96)	9.45 (4.80)	28.18 (1.72)	2.64 (0.67)

^a^K-MMSE: Korean Mini-Mental State Examination. A perfect score is 30.

^b^Response to the survey item “I have a swallowing problem” (with a scale of 0=never, 1=seldom, 2=sometimes, 3=often, and 4=always).

^c^N/A: not applicable.

### App Therapy Contents

The app contents included a swallowing monitoring and intervention protocol with 3 therapeutic methods or maneuvers: (1) effortful prolonged swallowing, which incorporated effortful swallowing [35] with the Mendelsohn maneuver [19,36], (2) the effortful pitch glide exercise [37], and (3) effortful tongue rotation [29]. The 3 effortful maneuvers were named A Successful Swallow with Effortful Trainings (ASSET). Methods for each training maneuver used in this study are shown in Table 2.

Participants were required to perform a total of 120 sessions (3 times/day, 5 days/week for 8 weeks). A 1-day session involved 20 repetitions of each of the 3 exercises (effortful prolonged swallowing, effortful pitch glide, effortful tongue rotation) in the morning, afternoon, and evening, with each time-specific session taking approximately 30 minutes. If the individuals failed to conduct the exercises on time, they were allowed to make up for the missed sessions on the same day only.

**Table 2:** Procedures for each training maneuver.

Exercise	Procedure
Effortful prolonged swallowing	(Phase 1: water swallow) Step 1. Hold 5 mL of water in your mouth. Step 2. Push all muscles around your neck and swallow as hard as you can (at this time, maintain your muscular strength for 2 seconds). (Phase 2: dry swallow) Step 1. Collect saliva in your mouth. Step 2. Push all muscles around your neck and swallow as hard as you can (at this time, maintain your muscular strength for 2 seconds).
Effortful pitch glide	Step 1. Make an elongated “ee” sound at a comfortable pitch. Step 2. Gradually raise your pitch to the highest pitch possible. Step 3. Keep the pitch at the highest range as long as possible.
Effortful tongue rotation	Step 1. Stretch your tongue and move it to one side of your cheek. Step 2. Rotate your tongue fully around the space between your teeth and back of the lips once while maintaining your strength for 5 seconds.

### App Features

We developed a tablet-based mHealth app (Android operating system) called 365 Healthy Swallowing Coach, which enables older adults to execute the given 3 types of swallowing training maneuvers without a clinician physically present. It contains an educational program, a feedback system, an adherence monitoring system, and a tailored training setting. The mobile app was designed to be downloaded and used on a tablet, the Samsung Galaxy Tab A (model No. SM-P580; Samsung), and to be user-friendly for older adults. Examples of user interface designs for older adults with decreased cognitive and psychomotor functions include (1) tabs arranged by use sequence for ease of navigation, (2) buttons containing both icons and text for ease of understanding, and (3) apposite button activation ranges, button sizes, and interbutton spaces empirically identified for the trade-off between ease of activation and prevention of inadvertent activation [34]. Furthermore, the mHealth app supports a variety of features that could enable older adults to properly and effectively train themselves. Examples of training compliance enablers include videos, animations, multimodal content, a biofeedback system for ease of understanding [38], and an automatic training data-logging system and training scheduler for ease of adherence monitoring [34]. A usability and feasibility test of the 365 Healthy Swallowing Coach app reported significantly higher usability scores from older adults with higher levels of education and smart device usage. Moreover, an increase in favorable responses regarding app acceptability, training program utilization, emotional responses, and learning experience with the intervention program was observed as the participants continued to use the app [34].

#### Educational Program

**Figure 1:**
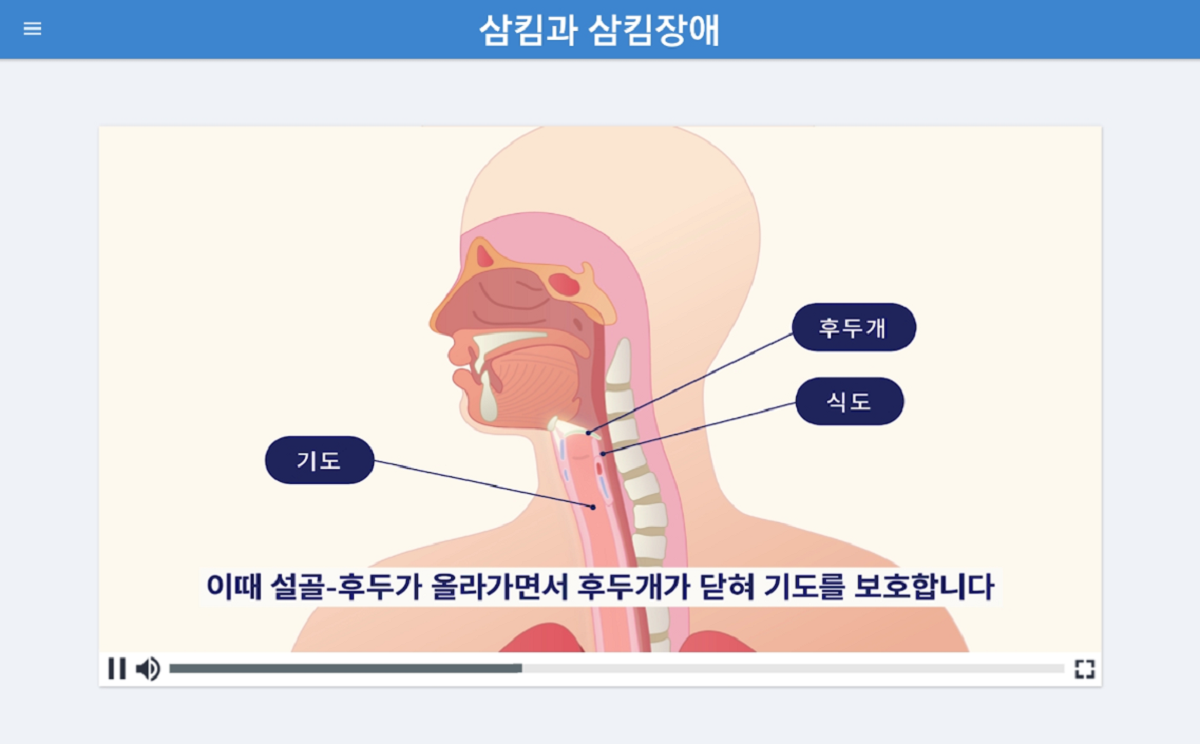
A screenshot of an example introductory video on the basics of swallowing mechanisms (describing hyolaryngeal excursion and epiglottic closure) and dysphagia. The caption in the video states, “At this point, the epiglottis closes as the hyoid bone and the larynx rise, protecting the airway.”

**Figure 2:**
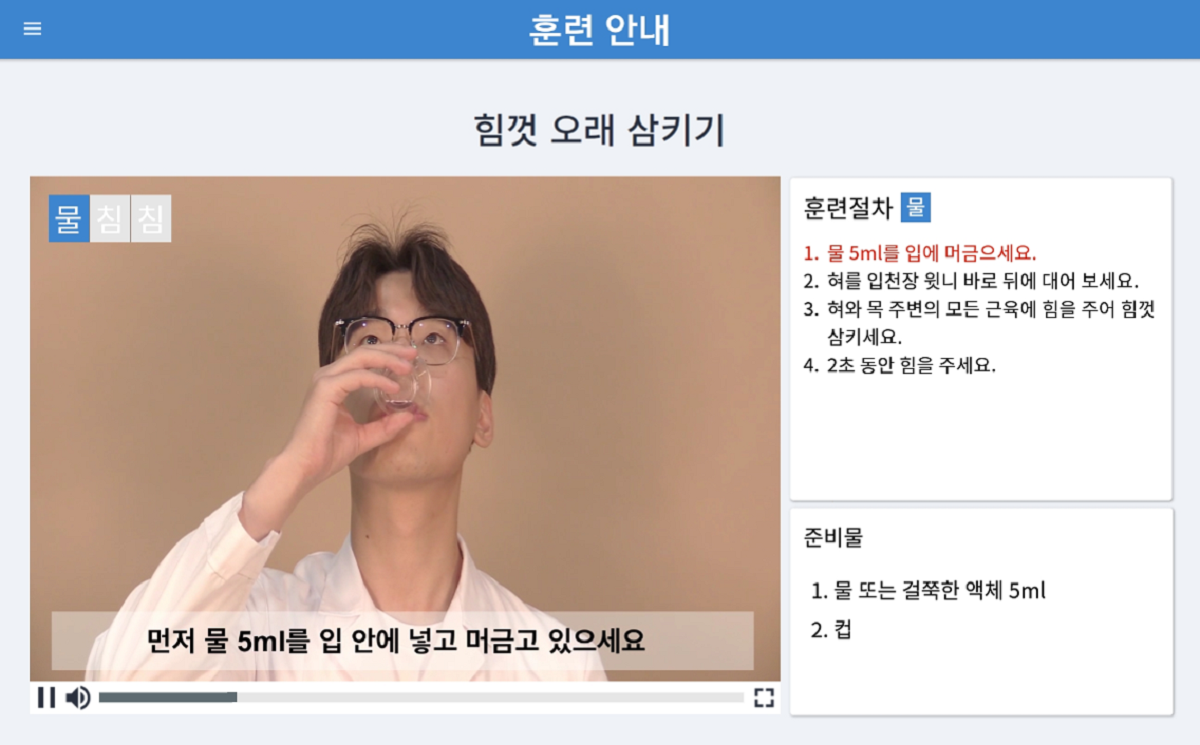
A screenshot of the educational demo video of the effortful prolonged swallowing exercise maneuver in the 365 Healthy Swallowing Coach app, with training procedures (top right) and preparation materials (bottom right). The caption in the video says, “First, hold 5 mL of water in your mouth.”

The educational content of the 365 Healthy Swallowing Coach app includes introductory videos on the basics of swallowing mechanisms and swallowing disorders, as well as training methods for each exercise (ie, effortful prolonged swallowing, effortful pitch glide, and effortful tongue rotation). Information on the basics of swallowing is introduced through a self-produced animation in the current study. The animation educates users on the physiological changes that occur during swallowing, such as epiglottic closure for airway protection, and promotes awareness on the danger of aspiration and pneumonia. The educational video for each exercise method consists of a demonstration carried out by a researcher and a practice trial. Users can practice each method after observing the demonstrations, which include a live-action video for effortful prolonged swallowing and effortful tongue rotation or a real-time pitch glide graph for effortful pitch glide. These videos are always shown prior to the beginning of the actual training sessions to ensure that users perform the exercises in an appropriate way. Screenshots of the educational content are shown in Figures 1 and 2.

#### Biofeedback System

**Figure 3:**
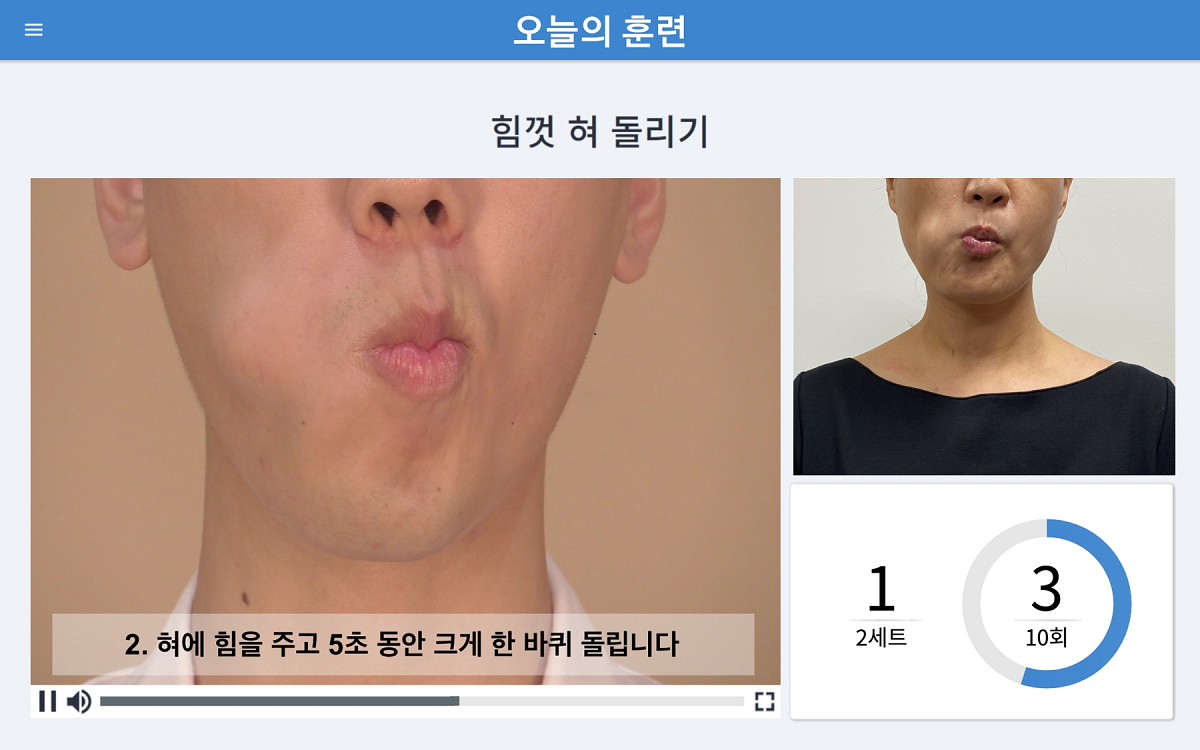
A screenshot of the visual biofeedback in the “Today’s Training” section, with the effortful tongue rotation demo video displayed on the left, the mirror function on the right, and the number of repetitions on the bottom right. The caption on the screen says, “2. Rotate your tongue fully around the space between your teeth and the back of the lips once while maintaining your strength for 5 seconds.”

**Figure 4:**
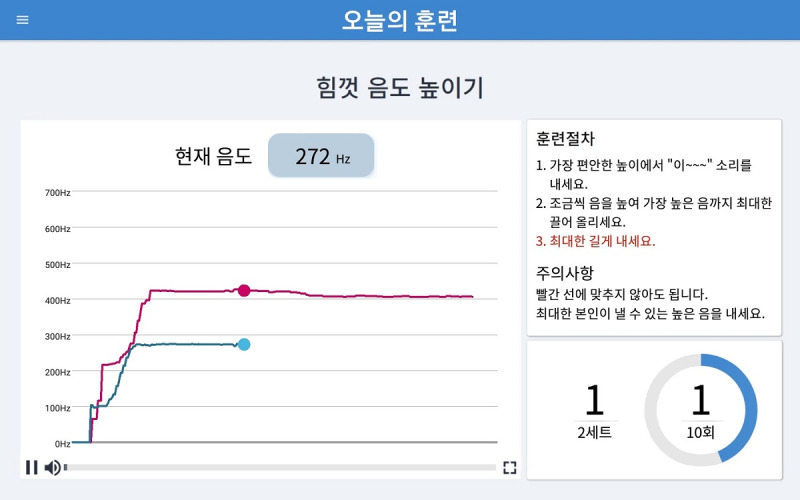
A screenshot of the visual biofeedback in the “Today’s Training” section during the effortful pitch glide maneuver, with a real-time pitch graph on the left, the training procedures on the right, and the number of repetitions indicated on the bottom right.

While the user exercises, the app provides instant visual biofeedback. For the effortful prolonged swallowing and effortful tongue rotation exercises, the screen displays a demo video and a mirror function on the same screen so that users can follow the demonstration while watching themselves train, as seen in Figure 3. The screenshot of the effortful pitch glide exercise displays a real-time pitch glide graph with 2 colors (Figure 4). The red line represents the pitch of the demonstration audio, and the blue line represents the user’s pitch. Users glide and adjust their pitch according to the shape of the red line. The biofeedback system allows users to appropriately perform the exercise methods each time by providing instant feedback regarding their performance.

#### Adherence Monitoring System

**Figure 5:**
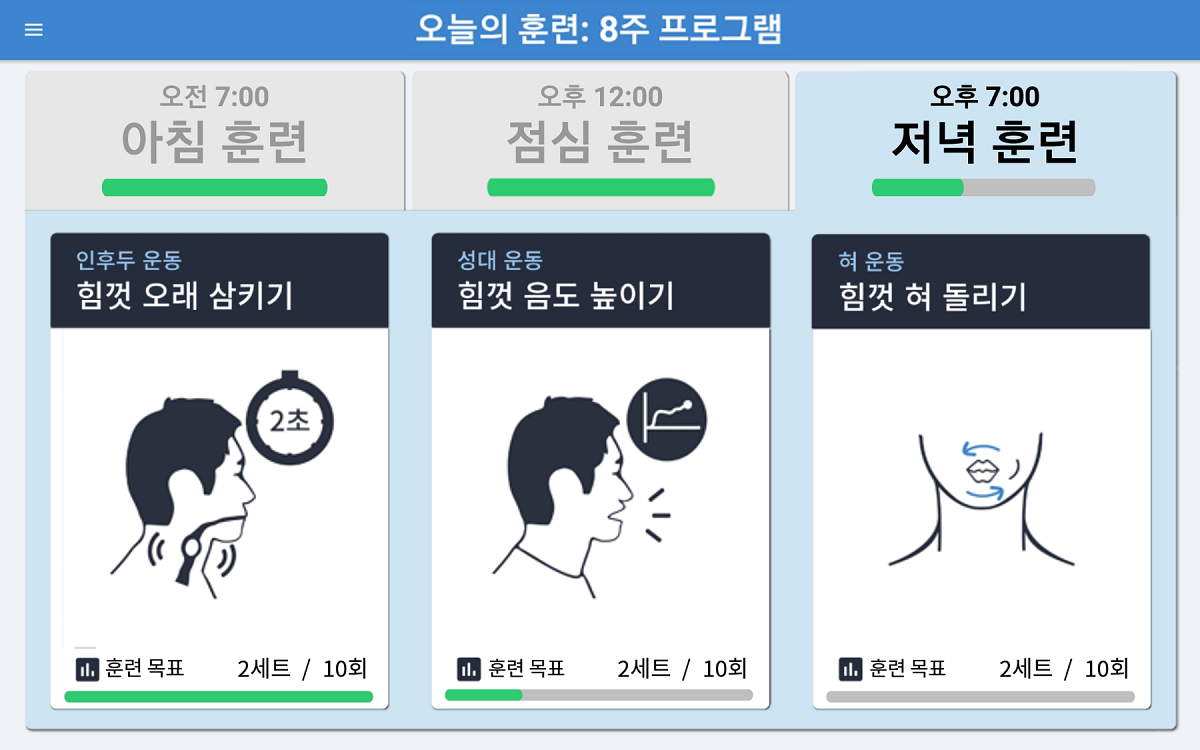
A screenshot of the exercise selection section (for the swallowing function training) of the 365 Healthy Swallowing Coach app, with green bars indicating exercise- and time-specific progress.

Users can monitor their progress through an automatic data-logging system that guides them through the performance of rigorous daily sessions. Figure 5 shows a screenshot of the exercise selection page for the swallowing function training. The exercise selection screen and the exercise record screen of the app show the time-specific and daily progress, respectively. The exercise selection screen initially shows empty progress bars on the selection tabs of each time-specific training session (ie, morning, afternoon, and evening training) on the top portion of the screen and on each exercise (ie, effortful prolonged swallowing, effortful pitch glide, and effortful tongue rotation) on the bottom portion of the screen (Figure 5). Meanwhile, the overall progress can also be checked on the exercise record screen, where a monthly calendar is provided, with the daily performance rate marked with a green (performance rate of 100%), orange (50% to 99%), or red (less than 50%) circle around a specific date. By using the adherence monitoring system of the mHealth app, users can constantly check their progress, which can help minimize the number of missed exercises.

### Procedures

**Figure 6:**
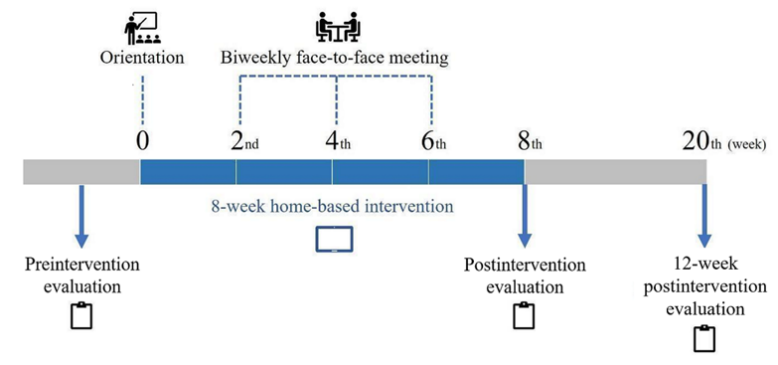
Flowchart of the 8-week home-based intervention and maintenance study.

A flowchart of the 8-week home-based intervention study with biweekly (ie, every 2 weeks) human mediation is presented in Figure 6. Two weeks before the beginning of the intervention program, participants’ demographic information and medical histories were collected, and their isometric and swallowing tongue pressures were measured by a clinician. Subsequently, they were educated on how to operate the designated tablet and the swallowing function training contents of the 365 Healthy Swallowing Coach app before starting their home training using the mobile device (orientation stage). Upon commencing the intervention program, the researchers held biweekly face-to-face meetings with each of the participants for the purpose of checking the participant’s ability to carry out the training sessions without assistance and perform the exercises in accordance with the given protocol. In this way, the researchers acted as evaluators who checked and monitored the training status of the participants. However, since the primary purpose of the face-to-face meetings was strictly confined to monitoring the participants’ progress, the researchers minimized giving advice that might directly influence their self-administered performance. Each face-to-face meeting was held for approximately 30 minutes. During the meetings, the participants were asked to navigate through the app (ie, from the main screen of the tablet to the training selection screen) and perform 1 training session as an example while the researchers monitored their presentation. If the participants failed to operate the app properly or performed training inaccurately, they were advised by the researchers to try to correct themselves. After each participant finished a training session and closed the app, the researchers then checked the participant’s progress by reviewing the sessions tracked in their tablet.

At postintervention (ie, immediately after the 8-week intervention), the participants’ isometric and swallowing tongue pressures were evaluated, as previously performed during the preintervention evaluation stage. No additional intervention or intervention-related mediation took place after the postintervention evaluation stage. Then, at 12 weeks postintervention (ie, 20th week from the beginning of the intervention program), the participants’ isometric and swallowing tongue pressures were reevaluated in an identical manner as in the preintervention and postintervention evaluation stages in order to evaluate the maintenance effect of the intervention program.

**Figure 7:**
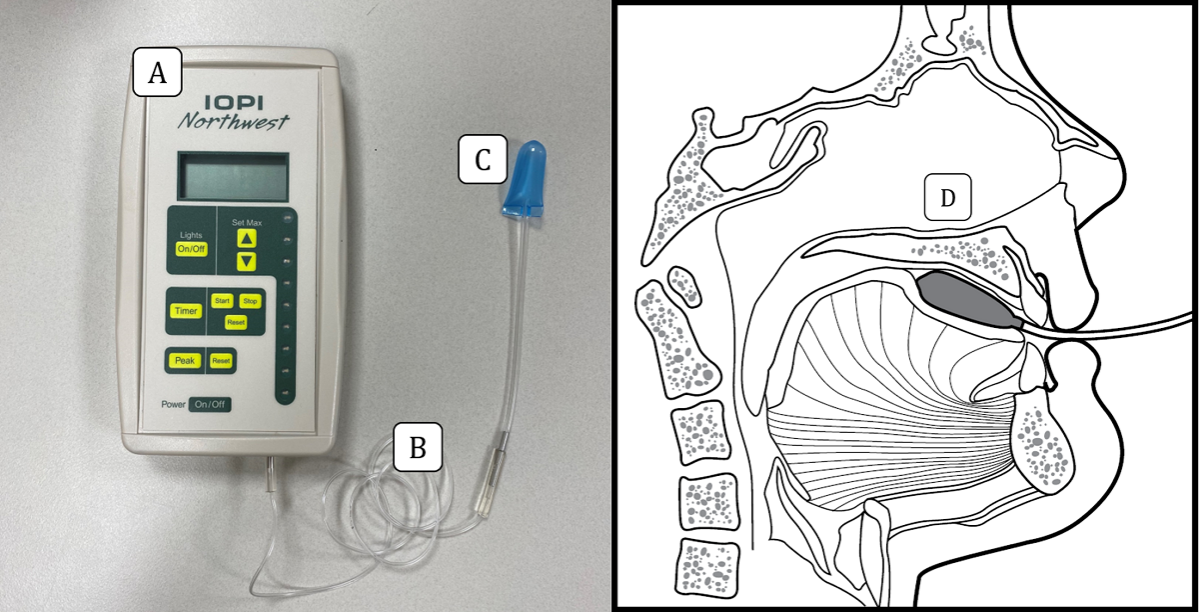
The Iowa Oral Performance Instrument components and the tongue bulb position. (A) Iowa Oral Performance Instrument model 2.1, (B) connecting tube, (C) air-filled tongue bulb, and (D) the position of the tongue bulb against the hard palate [39] (cited with permission).

Swallowing and isometric tongue pressures were measured using the Iowa Oral Performance Instrument (IOPI) (model 2.1; IOPI Medical LLC). The IOPI is a handheld device with a connecting tube and a measurement bulb (Figure 7) used to measure tongue pressure against the hard palate in an air-filled bulb.

The evaluations were conducted by the authors HK, N-BC, and SS. All participants had a verbal training session before having their perioral pressure recorded. During the tongue pressure measurement, the participants were in a comfortable and relaxed upright seated position to achieve a natural head posture, and the researchers oversaw their posture and the bulb position. When measuring the swallowing tongue pressure, the participants were asked to swallow the saliva as naturally as possible while the tongue bulb was placed between their tongue and the anterior one-third of their hard palate. When measuring the isometric tongue pressure, they were asked to push the bulb with maximum force toward their hard palate with their tongue for 2 seconds.

Each measurement was repeated 2 times. The pressure was recorded in kilopascals and the maximum value was retrieved. For swallowing tongue pressure, the participant was asked to perform a dry swallow [7]. Tongue pressure measurements were recorded before and after the intervention, as well as at 12 weeks postintervention. Immediate postintervention evaluation and 12-week postintervention evaluation findings were compared with preintervention evaluation findings to identify the short-term and long-term effects of the given therapy sessions. Statistical analysis was conducted using the Wilcoxon signed rank test (SPSS version 25; IBM Corp).

## Results

### Adherence to Intervention Program

The adherence of all 11 participants was evaluated at the end of the 8-week intervention program. Table 3 presents the adherence of each participant. Out of the 11 participants, 8 completed the 8-week program with the proper amount of training suggested by the researchers (adherence of 83.2%-100.8%) and were categorized as compliant participants. Only these participants were included in the data analysis. Meanwhile, 3 participants (No. 9, No. 10, and No. 11) labeled as noncompliant participants were excluded from the analysis due to failure to correctly adhere to the program protocol. Participants No. 9 and No. 10 were excluded because they completed only 35.4% and 41.1% of the required sessions, respectively. The 2 participants reported that they did not have enough time to carry out the training sessions and that they could not perform each training session in accordance with the given protocol due to the excessive amount of training. Meanwhile, participant No. 11 was excluded due to greatly exceeding (264.7%) the amount of training required. She reported that she did not understand the adherence monitoring system of the app due to unfamiliarity with the technology, resulting in an excessive amount of training.

**Table 3:** Adherence of each participant.

Participant classification and number			Adherence, %						
			Effortful prolonged swallowing		Effortful pitch glide		Effortful tongue rotation		Total
**Compliant participants**									
	1	93.0		93.7		92.9		93.2	
	2	101.3		98.7		100.0		100.0	
	3	97.5		97.4		97.4		97.4	
	4	85.3		82.2		82.1		83.2	
	5	101.0		100.3		97.2		99.5	
	6	93.8		91.6		89.6		91.7	
	7	96.3		93.7		95.7		95.2	
	8	100.1		101.1		101.3		100.8	
**Noncompliant participants**									
	9	34.9		37.0		34.2		35.4	
	10	48.5		37.8		37.1		41.1	
	11	252.7		305.2		236.3		264.7	

### Intervention Effect: Preintervention Versus Postintervention

The 8 participants who properly completed the 8-week training protocol, which was mediated by 3 sessions of biweekly meetings, demonstrated statistically significant increases in swallowing tongue pressure postintervention (median 26.5 kPa) compared with the preintervention (median 17.5 kPa) values (z=–1.992, *P*=.046). On the other hand, maximum isometric tongue pressures were not statistically different between the postintervention (median 41 kPa) and preintervention (median 40.5 kPa) evaluation (z=–0.632, *P*=.53). Table 4 and 5 show ranks and test statistics of swallowing and isometric tongue pressure before and after the intervention.

**Table 4:** Ranks and test statistics of swallowing tongue pressure before and after the intervention.

Swallowing tongue pressure		Participants, n	Pressure (kPa), mean (SD)	Pressure (kPa), range	Pressure (kPa), median	Mean rank	Sum of ranks	*P* value^a^	z^b^
**Intervention**								.046	–1.992
	Preintervention	8	18.3 (6.5)	7-27	17.5	N/A^c^	N/A		
	Postintervention	8	27.4 (9.4)	12-41	26.5	N/A	N/A		
**Rank: post-pre**								N/A	N/A
	Negative ranks	1^d^	N/A	N/A	N/A	1	1		
	Positive ranks	5^e^	N/A	N/A	N/A	4	20.5		
	Ties	2^f^	N/A	N/A	N/A	N/A	N/A		
	Total	8	N/A	N/A	N/A	N/A	N/A		

^a^Wilcoxon signed ranks test.

^b^Based on negative ranks.

^c^N/A: not applicable.

^d^Postintervention<preintervention.

^e^Postintervention>preintervention.

^f^Postintervention=preintervention.

**Table 5:** Ranks and test statistics of isometric tongue pressure before and after the intervention.

Isometric tongue pressure		Participants, n	Pressure (kPa), mean (SD)	Pressure (kPa), range	Pressure (kPa), median	Mean rank	Sum of ranks	*P* value^a^	z^b^
**Intervention**								.53	–0.632
	Preintervention	8	40.3 (5.1)	29-46	40.5	N/A^c^	N/A		
	Postintervention	8	41.0 (8.0)	29-52	41.0	N/A	N/A		
**Rank: post-pre**								N/A	N/A
	Negative ranks	3^d^	N/A	N/A	N/A	4.5	13.5		
	Positive ranks	5^e^	N/A	N/A	N/A	4.5	22.5		
	Ties	0^f^	N/A	N/A	N/A	N/A	N/A		
	Total	8	N/A	N/A	N/A	N/A	N/A		

^a^Wilcoxon signed ranks test.

^b^Based on negative ranks.

^c^N/A: not applicable.

^d^Postintervention<preintervention.

^e^Postintervention>preintervention.

^f^Postintervention=preintervention.

### Long-Term Effect: Preintervention Versus 12-Week Postintervention

Of the 8 participants, 6 (No. 1, No. 2, No. 3, No. 5, No. 7, No. 8) agreed to participate in the 12-week postintervention evaluation for examination of maintenance effects. Compared with preintervention (median 19 kPa), there was no statistically significant difference in swallowing tongue pressure at the 12-week postintervention (median 20.5 kPa) (z=–0.734, *P*=.46). Table 6 presents ranks and test statistics of swallowing tongue pressure measured at the preintervention and 12-week postintervention evaluation stages.

**Table 6:** Ranks and test statistics of swallowing tongue pressure before the intervention and 12 weeks after the intervention.

			Participants, n		Pressure (kPa), mean (SD)		Pressure (kPa), range		Pressure (kPa), median		Mean rank		Sum of ranks		*P* value^a^		z^b^
**Intervention**															.46		–0.734
	Preintervention	6		18.8 (7.5)		7-27		19.0		N/A^c^		N/A					
	12-week postintervention	6		24.8 (14.0)		12-45		20.5		N/A		N/A					
**Rank: 12-week post–pre**															N/A		N/A
	Negative ranks	3^d^		N/A		N/A		N/A		2.33		7					
	Positive ranks	3^e^		N/A		N/A		N/A		4.67		14					
	Ties	0^f^		N/A		N/A		N/A		N/A		N/A					
	Total	6		N/A		N/A		N/A		N/A		N/A					

^a^Wilcoxon signed ranks test.

^b^Based on negative ranks.

^c^N/A: not applicable.

^c^12-week postintervention<preintervention.

^d^12-week postintervention>preintervention.

^e^12-week postintervention=preintervention.

## Discussion

### Overview

To the best of our knowledge, this investigation is the first training study addressing an mHealth tongue-strengthening exercise for older adults. As tongue pressure measures have been determined to be effective indicators of swallowing function [7,8], an increase in tongue pressure may positively influence swallowing function as well. Decreases in swallowing tongue pressure generated during dry and high-viscosity liquid or bolus swallowing is prevalent in older adults [7,17]. Since such decline may be indicative of risk factors of dysphagia, looking at measures of swallowing tongue pressure in order to improve swallowing rehabilitation endeavors is a compelling pursuit [17]. In this study, a tablet-based swallowing training app with biweekly human mediation was used for 8 weeks by older adults who complained of swallowing difficulties. All 8 participants who completed the training demonstrated an increase in swallowing tongue pressure, even though therapists were not physically present during the training sessions. It should be noted that biweekly face-to-face meetings were held between the participants and the researchers for the main purpose of checking and monitoring the participants’ training progress, and this process may have affected the end results (ie, increased swallowing tongue pressure) of the intervention program. There are several important issues that must be recognized regarding these study findings.

### Exercise Maneuvers

Previous studies have shown that various tongue-strengthening exercises resulted in an increase in tongue strength and pressure [40-44]. Meanwhile, the current intervention contents (ASSET) adopted 3 therapeutic methods or maneuvers (ie, effortful prolonged swallowing, effortful pitch glide, and effortful tongue rotation) in combination, which led to positive effects on the swallowing tongue pressure of the participants, as indicated by the study results. However, several differences have been observed in treatment effects upon comparison of the current study to those that adopted a single therapy maneuver.

First, the current study implemented effortful prolonged swallowing, a combination of effortful swallowing training and the Mendelsohn maneuver training. In previous studies, training outcomes of effortful swallowing alone only revealed significant increases in the maximum isometric pressure of the anterior tongue, lingua-palatal pressures, and swallowing functions [41,45]. However, in this study, effortful prolonged swallowing positively affected swallowing tongue pressure. The functions of the extrinsic tongue muscles (ie, hyoglossus), which were shown to be active during the Mendelsohn maneuver [46], may have improved after therapy, thus benefiting the participants and positively affecting their swallowing tongue pressure.

Second, effortful tongue rotation is regarded as a promising training method, since swallowing tongue pressure is closely related to dysphagia symptoms such as aspiration [9] and oropharyngeal residue [47]. A previously conducted study observed a significant increase in maximum isometric tongue pressure after effortful tongue rotation [29]. Interestingly, however, only the swallowing tongue pressure and not the maximum isometric tongue pressure was enhanced in this study when the isokinetic effortful tongue rotation exercise was implemented.

Partial discrepancies observed among the previous and current results may be ascribed to the fact that the triple-combination therapy (ASSET) of effortful prolonged swallowing, effortful pitch glide, and effortful tongue rotation was employed in this study. Hence, the combination effect is not yet comparable to that of a single therapy maneuver. Consequently, the results from this study protocol are not indicative of the individual effect of each treatment method on posttreatment measures, since the treatment effects of each of these methods were not evaluated separately. Therefore, it cannot be concluded that the additive effect of the triple-combination therapy as a whole is equal or similar to the arithmetic addition of each individual exercise’s training effect. The effect that the 3 combined methods yield may be smaller than the effect that each method provides, or it may be greater [48] than the simple addition of individual effects.

### Optimal Dosage

Even though a significant effect on swallowing tongue pressure was obtained from this intervention program, it remains unclear if the actual cumulative exercise dosage (ie, intensity, frequency, and duration) employed in this study is significant [49]. According to a scoping review, a tongue exercise dosage of 3 times a day for 7 days with a frequency of 30 repetitions for each exercise was the most widely implemented regimen for studies including single tongue exercises [50]. However, for studies that adopted combined exercises (ie, more than one therapy method), treatment dosage, frequency, and duration varied widely, and thus, the improvements in various outcome measures could not be concluded to be attributable to the use of particular treatment dosages [50].

Determining the optimal exercise dosage of a treatment program is crucial, as overtraining may contribute to exercise-induced fatigue [51] and high dropout rates [52], whereas undertraining may not result in any benefit [53]. In fact, some participants in this study complained of the excessive training load and stated that they could not complete the intervention program.

The “active ingredients” of interventions need to be identified in order to determine the optimal intervention dosage and intensity [49]. The active ingredients are components in an intervention that can influence the treatment results. Client-related variables, including life situations or circumstances, or therapy-related variables, such as the complexity of treatments and the treatment duration [54], may also influence the optimal therapeutic dosages. Since the dosage stated in repetitions (eg, 20 exercise repetitions per session) indicates how many times an “active ingredient” appears per training session, it may be important to represent exercise dosage in such a form when determining the optimal intervention dosage for speech language pathology–related fields [49].

Choosing to adopt a distributed intervention or an intensive intervention is also critical in determining the ideal intervention dosage and intensity. Previous studies have adopted intervention programs for various speech and language disorders with different levels of intensity [55-57]. One study that compared the intervention results of a distributed (8 weeks, 6 hours per week, for a total of 48 hours) and intensive (3 weeks, 16 hours per week, for a total of 48 hours) aphasia intervention showed that the distributed intervention derived greater therapeutic gains compared with the intensive treatment [55]. The protocol duration implemented by this study is comparable to the distributed intervention adopted in the aforementioned study because the total intervention duration of this mHealth program was also 8 weeks (20 repetitions of each exercise, totaling 60 repetitions/session, 3 sessions/day, 5 times/week). However, the exact dose per session, stated as the number of repetitions, was not indicated in the aphasia intervention study. Thus, an exact one-to-one comparison is not possible.

### Long-Term Effects

The current study measured the swallowing tongue pressure at baseline and at 2 follow-up time points, including at 20 weeks after the intervention commenced (ie, 12-week postintervention), in order to investigate long-term intervention effects. Tracking changes in gained muscle (ie, tongue) pressure after the removal of an intervention is of particular interest, since a consideration of detraining effects may be necessary for the effective planning of aftercare [30]. In the current study, the training effect in the swallowing tongue pressure observed at the termination of the 8-week intervention program did not remain upon measuring it at 12 weeks postintervention. Despite the clinical implications of detraining effects, however, studies that examine the long-term effects of swallowing interventions are largely lacking [30,58]. Past studies show inconsistent results in posttreatment decay of lingual strength. Discrepancies in the findings from previous studies may be ascribed to several methodological differences, including (1) the postintervention measurement points, (2) the age of the participants, (3) the baseline health conditions of the participants, (4) the intervention delivery method, and (5) the type of outcome measure used.

The first factor is the postintervention measurement points. When the current results are compared with those of past studies, the long-term efficacy of tongue-strengthening exercises does not seem to show a consistent correlation with the measurement point alone. For instance, significant decreases in tongue strength have been observed after 2 to 4 weeks [30] and after 12 weeks (in the current study) of detraining, while no changes or decreases have been found at 4 weeks [59] and 28 weeks [40] postintervention. Since participant details and intervention dosages varied across the studies, the sole effect of postintervention measurement points needs to be addressed in a separate controlled study.

The second and third factors are the age and the baseline health conditions of the participants. A common observation is that older adults are more vulnerable to posttreatment decay [60,61]. Moreover, detraining effects are known to be larger for those who exhibit more impaired swallowing than those with more functional swallowing at baseline [58]. Hence, we hypothesize that the relatively high age and the swallowing difficulties of this study’s participants may have contributed to their markedly decreased posttreatment performance compared with the aforementioned studies, which targeted healthy adults without swallowing problems.

The fourth factor is the intervention delivery method. This study employed a tablet-based mHealth app for a swallowing intervention, which fundamentally differs from traditional methods, as telepractice requires participants’ self-management with the technology and their active engagement [62] for higher performance. Hence, it is possible that the long-term effects of telepractice interventions are susceptible to individual-dependent aspects (eg, technology learning rate, engagement rate).

The fifth factor is the type of outcome measure used (ie, isometric tongue pressure vs swallowing tongue pressure). As seen from the results, the comparison between isometric and swallowing tongue pressure demonstrated that only the latter was significantly improved in the posttreatment performance. In addition, both tongue-related measures returned to baseline after the detraining period, providing no direct clues regarding the correlation between a specific type of measure and its long-term effect. Although the maintenance of different types of measures is rarely found in the previous literature, the discrepancy between the posttreatment performance of isometric versus swallowing tongue pressure in this study indirectly suggests that the chosen outcome measure may be one possible factor that needs to be considered when interpreting long-term postintervention effects.

### mHealth and Self-Management

The mHealth platform can enhance the delivery of exercise maneuvers through improvements in training accuracy, economic efficiency, safety continuity, and self-management compared with traditional clinician-to-client intervention [63-66]. In this study, we used a biofeedback system involving a mirror function (in effortful prolonged swallowing and effortful tongue rotation) and a real-time pitch graph (in effortful pitch glide), preventing improper or incorrect performance, which may counteract the purpose of the maneuvers and become entrenched [67,68]. In this way, the training accuracy is maintained. Although there is no sufficient evidence that biofeedback benefits overall swallowing functions among patients with dysphagia, in studies that used tongue manometry as biofeedback, significant increases in maximum isometric and swallowing tongue pressure were observed in patients who received biofeedback compared with those who did not [69]. In addition, a previous study has suggested that home-based therapy can be as effective as office-based therapy if a biofeedback system is applied [70]. In addition to increasing training accuracy, the home-based mHealth training app may enable the participants to attend various training programs (eg, the 8-week program adopted in this study) without the burden of visiting clinics as frequently or the need to pay extra costs [71,72]. Thus, the time- and cost-effectiveness of mHealth programs with tongue-strengthening exercises may represent strong points for promoting their widespread use. Furthermore, older adults, including community-dwelling and patient groups, have higher risks for falls and disease complications, which highlights the need to promote their safety by reducing the frequency of their clinic visits [65,73]. The mHealth service could also allow for continuation of therapy sessions even during unexpected circumstances, such as pandemics (eg, COVID-19) [74]. A nondirect clinician-to-client intervention service could serve as a valid alternative to direct interaction.

In addition, it is highly necessary to construct mHealth tools that provide the support needed to promote participants’ adherence to the program. In general, the training adherence rate among the older population with various chronic diseases tends to decrease significantly for long-term training [75]. To address this, the tablet-based app used in this study included reminder functions, such as a calendar, a progress bar, and alarms, to promote participants’ self-monitoring of their adherence level. For instance, if a participant did not meet the training goal in a session, an incomplete red (or orange) circle would appear on the calendar and the progress bar would not be filled completely in green. The participant could then check their progress through visual biofeedback and finish the training accordingly. Such reminder features are commonly adopted in other training apps [76,77] and may be considered a crucial component of a self-therapy–related app. App features aimed at promoting users' adherence (eg, reminders, feedback), however, may not be designed to help users easily maintain long-term engagement without external support [78-80]. This is especially true for older adults who are not familiar with digital devices and apps, including mHealth devices [34,81]. Therefore, a hybrid method of intervention delivery (eg, mHealth app intervention with human mediation) may be necessary to help older adults maintain their adherence and thus potentially maximize the intended therapeutic effect, even when using the mHealth app as a primary means of swallowing health care. In this study, researchers checked the participants’ progress face-to-face once every 2 weeks to monitor their adherence to the intervention program using the mHealth app. A total of 8 of 11 participants successfully completed the program with optimal intended adherence, and it is possible that the overall adherence of the participants may have been lower in the absence of human mediation (ie, without the biweekly face-to-face meeting). A further study is warranted to see if similar therapeutic results would be observed for the mHealth intervention without external support.

Furthermore, in order to compensate for the possible issues caused by face-to-face interactions and to develop the current app platform into a more convenient means of intervention, it is suggested that real-time monitoring and control solutions be added into the app to allow clinicians to remotely keep track of the participants’ adherence and performance.

### Limitations and Future Directions

The findings from the study indicate that, using the mHealth app, the intervention was able to help older adults increase their swallowing tongue pressures. However, this study is not without limitations. This study involves a small number of participants, which does not permit the generalization of its findings. Accordingly, we could not classify participants by severity and make group comparisons. Furthermore, only 6 participants agreed to take part in the 12-week postintervention evaluation. The results from these participants may not have been sufficient to derive a generalized long-term effect of the given protocol. Thus, an implementation of the mHealth app with a larger sample size is proposed. Finally, although the effect of biweekly face-to-face monitoring on the increased swallowing tongue pressure measured at the postintervention phase is not negligible, the current study did not address the independent effect of the face-to-face monitoring on mHealth intervention effects.

We suggest additional issues that may be useful to address in future studies. First, although the participants of the current study did not receive any retraining during the 8 weeks of rest, it is likely that subsequent retraining may help participants retain their swallowing tongue pressures. Hence, a randomized controlled trial is further warranted to see if a treatment group (which follows a maintenance protocol after the intervention) exhibits higher long-term effects of training compared with a control group (which does not receive any retraining after the intervention). In addition, it may be necessary to take into consideration the frequency of postintervention measurements and the measurement points (eg, 8 weeks versus 12 weeks postintervention) to see if the detraining period influences the maintenance of swallowing functions.

Furthermore, the training intensity may have negatively affected the participants’ overall adherence. Several participants in our study expressed dissatisfaction toward the training intensity and frequency, claiming that 3 sessions a day is excessive, given that they have other plans during the day. Although the 365 Healthy Swallowing Coach app originally provided a tailored training setting for users (ie, users are able to customize the training schedule and training amount), this feature was made unavailable in the current study to measure the therapeutic effect of the fixed amount of training (ie, 3 times/day, 5 days/week). Hence, the reported problems related to the intensity of the protocol call for additional research to systematically investigate the optimal dosage of the mHealth swallowing intervention by comparing the effect of intensive (eg, 3 times/day, 7 days/week for 2 weeks) versus distributed (eg, 1 time/day, 3 days/week for 12 weeks) intervention methods.

### Conclusion

Swallowing tongue pressure is known to be closely related to dysphagia symptoms. This is the first study to demonstrate the effectiveness of the combined methods of effortful prolonged swallowing, effortful pitch glide, and effortful tongue rotation using mobile app training accompanied by biweekly human mediation in improving swallowing tongue pressure in older adults. The fixed amount of the training dosage (ie, 3 times/day, 5 days/week) was monitored biweekly to maintain the participants’ optimal adherence to the training program. As a result, a significant increase in swallowing tongue pressure was observed after 8 weeks of the intervention. This mHealth pilot study will help guide future intervention studies for older adults who have limited access to health care services. The mHealth app program is a promising method of providing therapy through the execution of intensive and repetitive tongue exercises for vulnerable older adults. In order to observe therapy efficacy with a larger sample size and to investigate long-term effects of the intervention program, further studies are warranted. Moreover, further studies using videofluoroscopic swallowing studies are needed to investigate the effects that the app-based therapy program may have on the various stages of swallowing.

## Abbreviations

ASSET: A Successful Swallow with Effortful Trainings
IOPI: Iowa Oral Performance Instrument
mHealth: mobile health
